# miR-190 promotes malignant transformation and progression of human urothelial cells through CDKN1B/p27 inhibition

**DOI:** 10.1186/s12935-021-01937-5

**Published:** 2021-04-29

**Authors:** Shirui Huang, Xiaohui Hua, Mengjiao Kuang, Junlan Zhu, Haiqi Mu, Zhongxian Tian, Xiaoqun Zheng, Qipeng Xie

**Affiliations:** 1 Department of Laboratory Medicine, The Second Affiliated Hospital & Yuying Children’s Hospital of Wenzhou Medical University, Wenzhou, 325035 Zhejiang China; 2 Department of Occupational Health and Environmental Health, School of Public Health, Anhui Medical University, Hefei, 230032 Anhui China; 3 School of Laboratory Medicine and Life Sciences, Wenzhou Medical University, Wenzhou, 325035 Zhejiang China

**Keywords:** miR-190, *TET1*, *CDKN1B*, Methylation, Bladder epithelial cell transformation

## Abstract

**Background:**

Although miR-190 has been reported to be related to human diseases, especially in the development and progression of cancer, its expression in human bladder cancer (BC) and potential contribution to BC remain unexplored.

**Methods:**

RT-qPCR was used to verify the expression level of miR-190 and CDKN1B. Flow cytometry (FCM) assays were performed to detect cell cycle. Soft agar assay was used to measure anchorage-independent growth ability. Methylation-Specific PCR, Dual-luciferase reporter assay and Western blotting were used to elucidate the potential mechanisms involved.

**Results:**

Our studies revealed that downregulation of the p27 (encoded by *CDKN1B* gene) protein is an important event related to miR-190, promoting the malignant transformation of bladder epithelial cells. miR-190 binds directly to *CDKN1B* 3’-UTR and destabilizes *CDKN1B* mRNA. Moreover, miR-190 downregulates TET1 by binding to the TET1 CDS region, which mediates hypermethylation of the *CDKN1B* promoter, thereby resulting in the downregulation of *CDKN1B* mRNA. These two aspects led to miR-190 inhibition of p27 protein expression in human BC cells. A more in-depth mechanistic study showed that c-Jun promotes the transcription of Talin2, the host gene of miR-190, thus upregulating the expression of miR-190 in human BC cells.

**Conclusions:**

In this study, we found that miR-190 plays an important role in the development of BC. Taken together, these findings indicate that miR-190 may promote the malignant transformation of human urothelial cells by downregulating *CDKN1B*, which strengthens our understanding of miR-190 in regulating BC cell transformation.

## Background

Bladder cancer (BC), the most frequent cancer of the urinary tract, is a highly prevalent deadly disease in developed countries. In 2018, there were approximately 81,190 new cases in the United States, of which 17,240 resulted in death [1]. Worldwide, the new incidence for BC is 549,393 and the total number of deaths is 199,922 [2]. At present, approximately 70% urothelial carcinomas (UC) are low-grade superficial papillary/non-muscle invasive tumors (NMIBC) with a relatively benign prognosis; the remaining 30% UCs are diagnosed as advanced muscle-invasive forms (MIBC) with poor outcomes [3–6]. Thus, elucidation of the mechanisms underlying the development and progression of BC is highly significant in improving diagnostic accuracy and clinical treatment.

MicroRNAs (miRNAs) are a type of non-coding RNAs of approximately 21 to 23 nt. They bind the 3’-untranslated region (UTR) to regulate the expression of most mRNAs and have important roles in the regulation of cellular processes [7, 8]. Therefore, the identification of miRNAs in human BC can provide valuable information for identifying new biomarkers for BC prognosis and/or new targets for treatment patterns. miR-190 is located in the intron region of the talin2 (TLN2) gene on chromosome 15q22.2 [9]. Previous studies have reported that miR-190 promotes hepatocellular carcinoma (HCC) cell proliferation and metastasis by acting as an oncogene, targeting PHLPP [10–12]. miR-190 also acts as a tumor suppressor in breast and gastric cancer [13, 14]. However, the expression and role of miR-190 in BC are rarely reported. We found that miR-190 expression was upregulated in human BCs by analyzing the TCGA database [15]. In the current study, we found that miR-190 promotes the malignant transformation of cells through the cyclin-dependent kinase inhibitor 1B (p27^Kip1^/p27), which is encoded by the *CDKN1B* gene in humans. It encodes a protein that belongs to the *Cip/Kip* family of cyclin-dependent kinase (Cdk) inhibitor proteins. The main function of p27 is to control the cell cycle progression at G1, stop or slow down the cell division cycle by inhibiting cyclin E-CDK2 or cyclin D-CDK4 complex activation [16]. We revealed that miR-190 directly binds to *CDKN1B* 3’-UTR and impairs its mRNA stability. In addition, miR-190-mediated hypermethylation of the *CDKN1B* promoter resulted in the downregulation of *CDKN1B* mRNA in human BC cells. The downregulation of the p27 protein is an important event related to miR-190 and promotes malignant transformation of BC cells. The results also showed that C-Jun promoted the transcription of TLN2, thereby upregulating the expression of miR-190 in human BC cells.

## Materials and methods

### Reagents, antibodies, and plasmids

TRIzol reagent (15596026) and SuperScript™ First-Strand Synthesis system (#1808-051) were purchased from Invitrogen (Grand Island, NY). Actinomycin D (sc-200906) was purchased from Santa Cruz Biotechnology (Dallas, TX, USA). The dual-luciferase assay kit was purchased from Promega (Madison, WI, E1960). The specific antibodies against DNMT3b (GTX129127), TET1 (GTX124207), TET2 (GTX124205), and GAPDH (GTX100118) were purchased from Genetex (Irvine, CA, USA) and antibodies specifically against p-c-Jun Ser73 (3270 S), c-JUN (9165 S), c-Jun(D) (5000 S), PARP (9542P), and Elk-1 (9182 S) were purchased from Cell Signaling Technology (Beverly, MA, USA). Antibodies specific for cyclin D1(sc-20044), DNMT3a (sc-373905), JunB (sc-46), CDK4 (sc-601), CDK6 (sc-7180), c-fos (sc-52), and Ets-1 (sc-55581) were obtained from Santa Cruz Biotechnology (Santa Cruz, CA, USA). Antibodies against β-Actin (66009-1-Ig) were purchased from Proteintech (Rosemont, IL, USA). V5-DEST-MIR190A and its control construct were kindly gifted by Dr. Ping-Yee Law (Department of Pharmacology, University of Minnesota, Minneapolis, MN, USA), and GFP–p27 was likewise a gift from Dr. Gustavo Baldassarre (Division of Experimental Oncology, Centro di Riferimento Oncologico, National Cancer Institute, Aviano, Italy). The human miR-190 inhibitor was purchased from GeneCopoeia™ Inc. (Rockville, MD, USA). The human p27 promoter (− 1324 to + 162) was cloned into the pGL3-basic luciferase reporter. Human *CDKN1B* and *TET1* 3′UTR were cloned into the pMIR-report plasmid. Plasmids were prepared using the Plasmid Preparation/Extraction Maxi kit (Qiagen, Valencia, CA, USA).

### Bioinformatics analysis

MiRNA expression data and corresponding clinical data for bladder cancer patients were obtained from The Cancer Genome Atlas (TCGA) data portal [TCGA Data Portal]. Normalized miRNA expression data were collected from the TCGA Data Portal using the Arraytool. Two different online tools, miRDB (http://www.mirdb.org/) [17] and Target scan (http://www.targetscan.org/vert_72/) [18] were used to identify target genes of miR-190.

### Cell cycle analysis

Constructed transfectants were cultured in each well of six-well plates to 70–80% confluence using normal culture medium. The cell culture medium was replaced with 0.1% FBS DMEM with 2 mmol/L L-glutamine and 25 µg gentamicin, and the constructed transfectants were again cultured for 24 h. Cells were then suspended in 70% ethanol for 24 h at 4 °C, then incubated with RNase A at 37 °C for 30 min, and stained with propidium iodide (PI) at 4 °C for 30 min. DNA content was determined by flow cytometry using the Epics XL flow cytometer (Beckman Coulter Inc., San Diego, CA), as previously described [19].

### Cell culture and transfection

UROtsa was cultured in 1640 with 10% FBS and UMUC3 was cultured in DMEM with 10% FBS. Cells were transfected with plasmid DNA using PolyJet™ DNA In Vitro Transfection Reagent (SignaGen Laboratories, Gaithersburg, MD, USA). Stable transfectants were selected with the corresponding antibiotics for 3–4 weeks, depending on the different transfected antibiotic resistance plasmids.

### Dual‐luciferase reporter assay

The dual luciferase assay kit was purchased from Promega (Madison, WI, USA). BC cells were co-transfected with either the TLN2 and *CDKN1B* promoter-luciferase reporter constructs or *CDKN1B* and TET1 3′-UTR-luciferase reporter constructs, together with the Renilla luciferase vector pRL-TK. After stabilization, the cells were treated with passive lysis buffer according to the dual-luciferase assay manual and then measured with a luminometer (Lumat LB9507, Berthold Tech., Bad Wildbad, Germany). For each analysis, the firefly luciferase signal was normalized to the Renilla luciferase signal to eliminate the difference in transfection efficiency, as previously described [20].

### Reverse transcription-qPCR (RT-qPCR)

Total RNA from the cells was isolated using the trizol reagent. Total RNA (5 µg) was then used for reverse transcription with oligo dT primer through the SuperScript™ First-Strand Synthesis system IV (Invitrogen, Grand Island, NY). Specific primer pairs were designed to amplify human CDKN1B (forward: 5′-CAA GTA CGA GTG GCA AGA G-3′, reverse: 5′-ATG CGT GTC CTC AGA GTT AG -3′) and GAPDH (forward: 5′-AGA AGG CTG GGG CTC ATT TG-3′, reverse: 5′-AGG GGC CAT CCA CAG TCT TC-3′). Total miRNAs were extracted using the miRNeasy Mini Kit (Qiagen, Valencia, CA, USA). Total RNA (1.0 µg) was used for reverse transcription following the manufacturer’s instructions, and miRNA expression was determined by the Q6 real-time PCR system (Applied Biosystems, Carlsbad, CA, USA) using the miScript PCR Starter Kit and miScript PCR kit II RT Kit (Qiagen, Valencia, CA, USA). U6 was used as the endogenous normalizer. The primer for miR-190 (5′- TGA TAT GTT TGA TAT ATT AGG T-3′) was synthesized by Genewiz Biotechnology (South Plainfield, USA). The cycle threshold (CT) value was measured, and the relative expression of mRNA was calculated based on the value of 2^−ΔΔCT^ as described in our published studies [21].

### Immunoblotting assay

Whole cells were washed twice with ice-cold PBS and then extracted using cell lysis buffer (10 mM pH7.4 Tris-HCl, 1% SDS, 1mM Na_3_VO_4_, and proteasome inhibitor) on ice. The materials were heated at 100 °C for 10 min and then ultrasonicated to destroy all nucleic acids. The protein concentration was measured using a NanoDrop 2000 spectrophotometer (Thermo Scientific, Holtsville, NY, USA). The cell extracts were subjected to Western blot analysis with each antibody. The protein bands specifically binding to the primary antibodies were detected using an alkaline phosphatase (AP) conjugate secondary antibody and enhanced chemifluorescence (ECF) Western blot analysis system (Amersham Pharmacia Biotech, Piscataway, NJ) as previously described [21]. The results shown are from at least three independent experiments.

### Anchorage‐independent growth

In brief, 1 × 10^4^ UROtsa (miR-190), UMUC3 (miR-190 inhibitor), UROtsa (miR-190/GFP-p27), and UMUC3 (miR-190 inhibitor/shp27) stable transfectants and control vector transfectants were exposed to Basal Medium Eagle (BME) containing 0.33% agar and seeded on the bottom layer of 0.5% agar in 10% FBS BME in each well of six-well plates. The cultures were maintained at 37 °C in a 5% CO2 incubator for 3–4 weeks, and the cell colonies with > 32 cells were scored. Colonies were observed and counted under a microscope (DMi1, Leica, Germany). The results are presented as the mean ± SD of colony number per 10,000 seeded cells in soft agar as described in a previous paper [22].

### DNA extraction, bisulfite DNA modification, and methylation‐specific PCR

CpG islands were predicted using MethPrimer 2.0 (http://www.urogene.org/ methprimer2/) for the upstream region of the *CDKN1B* Promoter. Genomic DNA from UROtsa and UMCU3 cells was extracted using a DNeasy Blood and Tissue Kit (# 69504, Qiagen, Valencia, CA, USA). Sodium bisulfite modification of DNA and subsequent purification was performed according to the manufacturer’s instructions for bisulfite conversion of unmethylated cytosines in DNA (EpiTect Bisulfite kit; #59104, Qiagen, Valencia, CA, USA).

Then, the bisulfite-treated genomic DNA was optimized for a methylation-specific PCR protocol, that is, 20 µL reactions containing 10 ng template, 10 µL 2× EpiTect Master Mix (Qiagen, Valencia, CA, USA), and 0.4 µM of a given set of methylation primers (Methylation-F, GTA GAT TAC GAG GTG GGG GTC; and Methylation-R, CTA AAA CGA AAC CTA AAA TTC GAA) or 0.4 µM each unmethylation primers (Unmethylation-F, TAG ATT ATG AGG TGG GGG TTG T; and Unmethylation-R, CCT AAA ACA AAA CCT AAA ATT CAA A). Touchdown PCR was then performed as follows: 95 °C for 10 min followed by five cycles of 94 °C for 30 s, 70 °C for 30 s, 72 °C for 30 s; five cycles of 94 °C for 30 s, 65 °C for 30 s, 72 °C for 30 s; and 30 cycles of 94 °C for 30 s, 60 °C for 30 s, 72 °C for 30 s. The final extension was performed at 72 °C for 7 min. All products were then separated on 2% high-resolution agarose gels and visualized by ethidium bromide staining. All PCR products were run in duplicate using EpiTect PCR Control DNA Set (#59695, Qiagen, Valencia, CA, USA).

### Statistical analyses

Student’s t-test was used to determine significant differences between the treated and untreated groups. Results are expressed as the mean ± SD from at least three independent experiments. *P* < 0.05 was considered to be a significant difference between the compared groups. All data were analyzed using GraphPad Software 6.0 (La Jolla, CA, USA).

## Results

### miR-190 was overexpressed in human BC tissues and contributed to bladder urothelial cell transformation

**Fig. 1:**
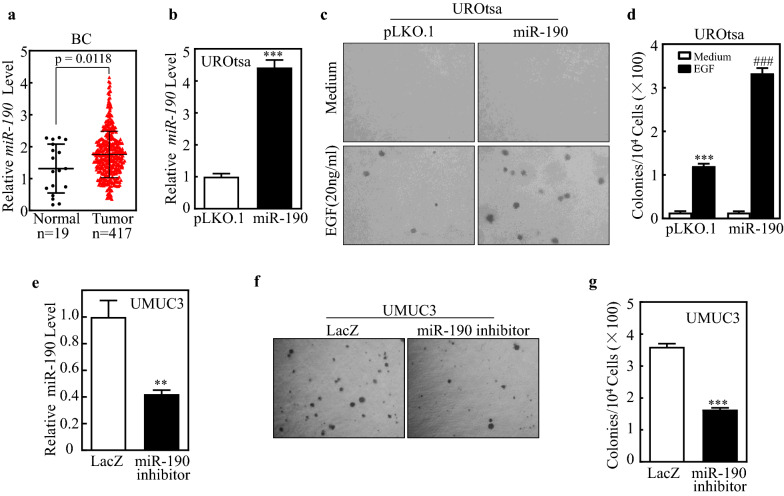
The expression of miR-190 in Human BC tissues and its role in bladder urothelial cell transformation. **a** The expression of miR-190 in BC tissues was compared with normal bladder tissues and miR-190 was up-regulated in BC tissues (p = 0.0118). **b** miR-190 and control vector were stably transfected into UROtsa. The symbol (*) indicates a significant increase (*P < 0.05, **P < 0.01, ***P < 0.001). **c**,** d** 1 × 10^4^ cells of UROtsa(pLKO.1) and UROtsa(miR-190) were subjected to soft agar assay in the presence of EGF (20 ng/ml). The images were captured under inverted microscopies after being incubated in a 37 °C with 5% CO_2_ incubator for 3 weeks (**c**) and the colonies were also counted (**d**). Each bar indicates the mean ± SD from triplicate assays. The symbol (*) indicates a significant increase as compared with the medium control, while the symbol (#) indicates a significant increase in comparison to UROtsa(pLKO.1) cells (*P < 0.05, **P < 0.01, ***P < 0.001; ^#^P < 0.05, ^##^P < 0.01, ^###^P < 0.001). **e **miR-190 sponge inhibitor (miR-190 inhibitor) and control vector were stably transfected into UMUC3 cells, and the symbol (*) indicates a significant decrease (*P < 0.05, **P < 0.01, ***P < 0.001). **f**,** g** the indicated cell transfectants were subjected to soft agar assay same as described in “**c**, **d**” (**f**, **g**); The symbol (*) indicates a significant decrease as compared with the UMUC3(LacZ) cells (*P < 0.05, **P < 0.01, ***P < 0.001)

We used the TCGA database to analyze the expression of miR-190 in 417 BC tissues vs. 19 normal bladder tissues. The results showed that miR-190 was upregulated in BC tissues (Fig. 1a). The levels of miR-190 were also assessed in human BC cell lines (T24 and UMUC3) and normal urothelial cells (UROtsa). The miR-190 expression in T24 and UMUC3 cells was significantly higher than that in UROtsa cells [23]. These results indicate that miR-190 plays an important role in the development of BC. To evaluate the effects of miR-190 on the development of human BC, miR-190 was stably transfected into UROtsa cells. The stable transfectants vector control UROtsa (pLKO.1) and miR-190 overexpression UROtsa (miR-190) were established and identified by qPCR, miR-190 level was significantly higher in UROtsa (miR-190) than that in its control UROtsa (pLKO.1) cells (Fig. 1b). EGF has been reported as a common tumor promoter in many experimental systems, such as human urothelial cells [24, 25]. We next used EGF to establish an EGF-induced cell malignant transformation experimental system, and then evaluated the ability of miR-190 to transform UROtsa cells. The results showed that overexpression of miR-190 led to a profound increase in anchorage-independent growth upon EGF exposure in comparison to that in UROtsa (pLKO.1) cells under the same experimental conditions (Fig. 1c, d), suggesting that miR-190 has a potential promoting effect on cell malignant transformation. To elucidate the role of miR-190 in BC cell lines, we inhibited miR-190 expression in UMUC3 cells, which have a high level of miR-190. The stable transfectants UMUC3 (LacZ) and UMUC3 (miR-190 inhibitor) were established and identified by qPCR, miR-190 level was significantly inhibited in UMUC3 (miR-190 inhibitor) compared with its control UMUC3 (LacZ) cells (Fig. 1e). The results from soft agar assay indicate that the anchorage-independent growth ability significantly decreased in UMUC3 (miR-190 inhibitor) cells compared to UMUC3 (LacZ) cells (Fig. 1f, g). These results suggest that miR-190 serves as a potential oncogene responsible for promoting human bladder urothelial cell transformation.

### Down-regulation of p27 plays a crucial role in miR-190’s induction of the S phase in cell cycles of BC cells

**Fig. 2:**
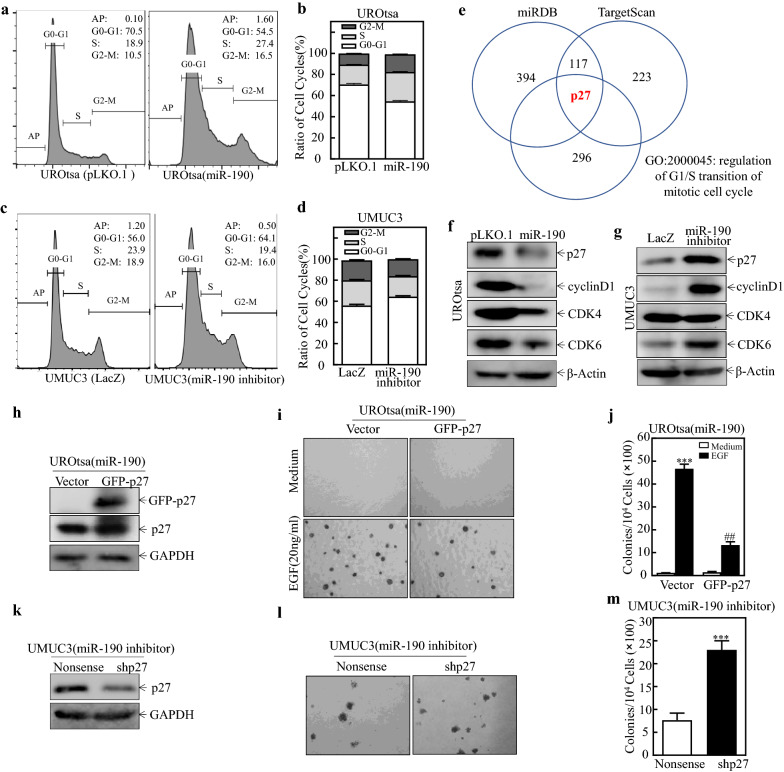
miR-190 induced BC cell growth by down-regulating p27 and promoting G1/S phase transition. **a–d** The indicated cells were seeded into 6-well plates and cultured to 70–80% confluence; after synchronization in 0.1% FBS medium for 24 h., cells were cultured in complete medium for another 24 h. and then subjected to cell cycle analysis by flow cytometry as described in Materials and Methods. **e** GO analysis of potential miR-190 targeted genes. **f, g, h, k** Cell lysates from the indicated cells were evaluated for p27, cyclin D1, CKD4, and CKD6 expression via western blots. β-Actin or GAPDH served as the loading control. (**i, j, l, m**) A soft agar assay was used to determine the effect of p27 overexpression (i, j) or knockdown (l, m) on anchorage-independent growth compared with vector control cells; Representative images of colonies from the indicated cells were captured under microscopy after 3 weeks of incubation. The number of colonies was counted under microscopy and the number of colonies was counted and presented as colonies per 10^4^ cells. The symbol (*) indicates a significant difference (*P < 0.05, **P < 0.01, ***P < 0.001; ^#^P < 0.05, ^##^P < 0.01, ^###^P < 0.001)

To determine the mechanism(s) behind miR-190’s promotion of BC cell transformation, the effect of miR-190 on the regulation of cell cycle progression was determined by flow cytometry in UROtsa and UMUC3 cells. As shown in Fig. 2a–d, miR-190 induced a significant G1/S phase transition, suggesting that the G1/S phase transition might be associated with the promoting effect of miR-190 on human BC cells. We used miRBD and TargetScan databases to analyze the potential target genes of miR-190. A total of 117 genes overlapped in the two databases. GO analysis showed that only CDKN1B/p27 was associated with the G1/S transition (Fig. 2e). We compared the expression levels of essential proteins related to the G1/S transition between UROtsa (pLKO.1) and UROtsa (miR-190), as well as between UMUC3 (LacZ) and UMUC3 (miR-190 inhibitor) cells. As shown in Fig. 2f, g, p27, cyclin D1, and CDK6 protein expression was profoundly decreased in UROtsa (miR-190) cells compared to that in UROtsa (pLKO.1) cells. As expected, p27, cyclin D1, and CDK6 was also markedly upregulated in UMUC3 (miR-190 inhibitor) compared with UMUC3 (LacZ) cells. Other cell cycle-related protein such as CDK4 does not meet the corresponding trend. Considering the fact that only p27 is a tumor suppressor gene and the result of the above-mentioned bioinformatics analysis, we thought that p27 may be a downstream effector of miR-190 and negatively regulates the transformation ability of BC cells.

To determine whether p27 was responsible for the promoting effect of miR-190 on BC cell transformation and anchorage-independent growth, UROtsa (miR-190) cells were stably transfected with a GFP-tagged p27 to restore p27 expression (Fig. 2h). As shown in Fig. 2i, j, ectopic expression of GFP-p27 markedly decreased EGF-induced anchorage-independent growth induced by miR-190, compared with that in scramble control vector cells. UMUC3 (miR-190 inhibitor) cells were also stably transfected with specific shRNA to knock down p27 expression (Fig. 2k). As shown in Fig. 2l, m, p27 knockdown profoundly increased anchorage-independent growth inhibited by the miR-190 inhibitor, compared with that in scramble control vector cells. These results indicate that overexpression of p27 reverses the promotion of miR-190 in BC cells. Overall, the above data indicate that the p27 protein downregulation is one of the important events associated with miR-190 in BCs in terms of promoting the malignant transformation of cells.

### miR-190 targeted CDKN1B mRNA 3’-UTR and downregulated its mRNA stability in human BC cells

**Fig. 3:**
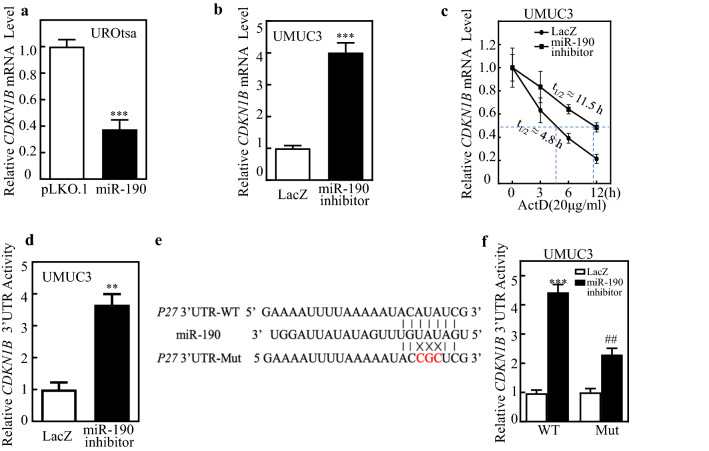
miR-190 reduces p27 mRNA stability by binding to its 3′UTR. **a**, **b** real-time PCR was performed to determine *CDKN1B* mRNA expression levels in UROtsa(miR-190) and UMUC3(miR-190 inhibitor) and their vector control cells. *GAPDH* was used as an internal control. Bars represent mean ± SD from three independent experiments. The symbol (*) indicates a significant difference (*P < 0.05, **P < 0.01, ***P < 0.001). **c** UMUC3(LacZ) and UMUC3(miR-190 inhibitor) cells were treated with Act D (20 µg/mL) for the indicated time periods. Total RNA was isolated and subjected to real-time PCR analysis for *CDKN1B* mRNA expression. **d** The *CDKN1B* 3′UTR reporters were co-transfected with pRL-TK into the indicated cells. Twenty-four hours post-transfection, the transfectants were extracted for determination of the luciferase activity, and TK was used as the internal control. The results are shown as *CDKN1B* 3’UTR activity relative to vector control transfectant, and each bar indicates the mean ± SD from three independent experiments. The symbol (*) indicates a significant difference (*P < 0.05, **P < 0.01, ***P < 0.001). **e** Potential miR-190 targeting sequences of the *CDKN1B* mRNA 3′-UTR were analyzed using TargetScan software. Schematics of the *CDKN1B* mRNA 3′-UTR luciferase reporter and its mutants (MUT) are shown. **f** WT and mutant *CDKN1B* 3′-UTR reporters were co-transfected with pRL-TK into the indicated UMUC3 (miR-190 inhibitor) transfectants. At 24 h after transfection, transfectants were extracted to assess luciferase activity; TK was used as the internal control. Each bar indicates the mean ± SD of three assays; *significant difference (*P < 0.05, **P < 0.01, ***P < 0.001; ^#^P < 0.05, ^##^P < 0.01, ^###^P < 0.001)

miRNAs play biological roles by modulating target gene expression through binding to the 3′-UTR of target genes, thereby causing changes in mRNA stability or protein translation inhibition [26]. To investigate the molecular mechanism underlying miR-190 regulation of *CDKN1B*, qPCR was performed to examine mRNA expression (Fig. 3a, b). The results showed that *CDKN1B* mRNA level was significantly lower in UROtsa (miR-190) than that in UROtsa (pLKO.1) cells; on the other hand, *CDKN1B* mRNA was profoundly higher in UMUC3 (miR-190 inhibitor) than that in UMUC3 (LacZ) cells, indicating that miR-190 suppresses *CDKN1B* at mRNA levels. The mRNA regulation includes transcription and mRNA stability. Therefore, we first examined the stability of *CDKN1B* mRNA. Upon treatment with actinomycin D (Act D), *CDKN1B* mRNA degradation rates in UMUC3 (miR-190 inhibitor) cells were much lower than those in UMUC3 (LacZ) cells (Fig. 3c), revealing that miR-190 inhibition stabilizes *CDKN1B* mRNA in human BC cells. Furthermore, the CDKN1B mRNA 3′-UTR activity of UMUC3 (miR-190 inhibitor) cells was significantly higher than that of UMUC3 (LacZ) cells (Fig. 3d), suggesting that miR-190 negatively modulates the stability of CDKN1B mRNA by acting on the 3′-UTR of CDKN1B. To determine whether the effect of miR-190 on *CDKN1B* mRNA stability suppression was due to its specific binding to a potential binding site in *CDKN1B* mRNA 3′-UTR, *CDKN1B* mRNA 3′-UTR-driven luciferase reporter (WT), and *CDKN1B* mRNA 3′-UTR mutant luciferase reporter (MUT) constructs were generated using the pMIR-Report luciferase vector, as shown in Fig. 3e. WT and mutant *CDKN1B* 3′-UTR luciferase reporters, with pRL-TK, were transiently transfected into UMUC3 (LacZ) and UMUC3 (miR-190 inhibitor) cells. As shown in Fig. 3f, the miR-190 inhibitor significantly increased the *CDKN1B* 3′-UTR WT luciferase reporter activity, whereas mutation of the miR-190-binding site at the *CDKN1B* mRNA 3′-UTR impaired the miR-190-inhibition-mediated increase in *CDKN1B* 3′-UTR luciferase reporter activity. This result indicates that miR-190 directly binds to the *CDKN1B* 3′-UTR and regulates its mRNA stability.

### miR-190 simultaneously inhibits CDKN1B transcription by upregulating its promoter methylation

**Fig. 4:**
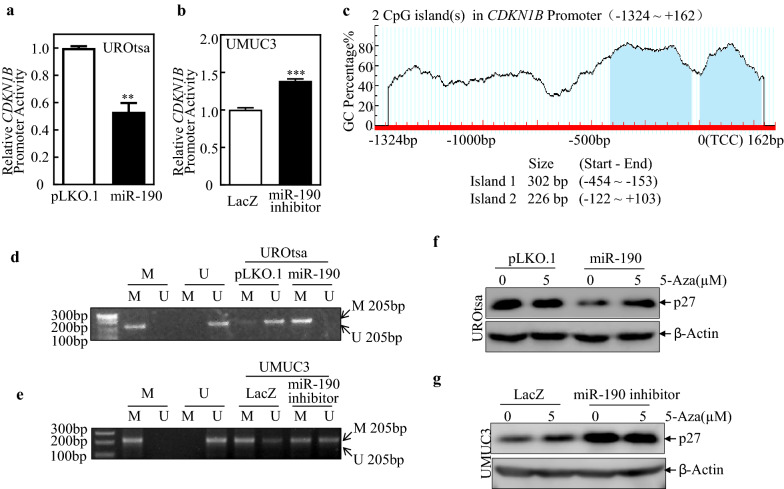
miR-190 inhibits ***CDKN1B*** transcription by up-regulating its promoter methylation.  **a**,** b** Indicated cells were transiently transfected with a *CDKN1B* promoter-driven luciferase reporter together with pRL-TK. Transfectants were seeded into 96-well plates to determine *CDKN1B* promoter transcriptional activity. pRL-TK was used as the internal control to normalize transfection efficiency. Bars indicate means ± SD from three replicate assays. The symbol (*) indicates a significant difference (*P < 0.05, **P < 0.01, ***P < 0.001). **c** Potential CpG Islands of human *CDKN1B* promoter were analyzed via MethPrimer v1.1beta software. **d**,** e** The methylation status of the *CDKN1B* promoter in the methylation region was determined using the MS-PCR assay in the indicated cells. A primer set was used to evaluate the methylated (M) and unmethylated (U) copies of *CDKN1B* promoter in the methylation region. Methylated control was used as positive control (M), whereas unmethylated control was used as negative control (U). **f**,** g** Indicated cells were pre-treated with 5-aza-2′-deoxycytidine and the cells were then subjected to Western Blot to analyze p27 protein level. β-Actin was used as a protein loading control

The level of *CDKN1B* mRNA in UMUC3 (miR-190 inhibitor) is up to 4 times higher than that in UMCU3 (LacZ) cells, and the half-life of *CDKN1B* mRNA is increased by approximately half. This led us to explore the possibility that *CDKN1B* is regulated at the transcription level. Therefore, we next transfected human *CDKN1B* promoter (from − 1324 to + 162)-driven luciferase reporters into UROtsa (miR-190), UMUC3 (miR-190 inhibitor), and their control transfectants. The results showed that overexpression of miR-190 decreased *CDKN1B* promoter-driven reporter transcription activity (Fig. 4a), and that inhibition of miR-190 increased *CDKN1B* promoter-driven reporter transcription activity (Fig. 4b), revealing that miR-190 also inhibits *CDKN1B* mRNA transcription. The CpG-rich region in the promoter region of cancer-related genes can be hypermethylated by epigenetic modification, which is an important mechanism for human cancer development [27]. To investigate whether miR-190 inhibited the *CDKN1B* transcription due to its promoter hypermethylation, we bioinformatically analyzed the potential CpG island of the human *CDKN1B* promoter (Fig. 4c) using the MethPrimer v1.1 beta database and then assessed the methylation of *CDKN1B* promoter from − 1324 to + 162 [28, 29]. The results showed that the *CDKN1B* promoter contains two CpG islands with sizes of 302 bp (-454 to -153) and 226 bp (-122 to + 103). To verify the methylation level of the *CDKN1B* promoter region, two independent predicted primer sets were used in the methylation-specific PCR (MS-PCR) system to amplify the methylated and unmethylated regions. As shown in Fig. 4d, the overexpression of miR-190 upregulated methylated DNA (M), resulting in a 205 bp band, accompanied by downregulation of unmethylated DNA (U), producing a 205-bp band, whereas the inhibition of miR-190 downregulated methylated DNA (M) accompanied by upregulation of unmethylated DNA (U) (Fig. 4e). To further investigate the role of promoter hypermethylation in miR190-mediated *CDKN1B* transcription downregulation, 5-aza-2′-deoxycytidine (5-Aza), a DNA methyltransferase (DNMT) inhibitor, was used to inhibit genomic DNA methylation in human BC cells [30]. The results showed that 5-Aza treatment reversed the miR-190 inhibition on the p27 protein (Fig. 4f, g). These results strongly suggest that miR-190-mediated *CDKN1B* promoter hypermethylation leads to *CDKN1B* mRNA downregulation in human BC cells.

### miR-190 downregulated TET1 by binding its CDS region and mediated CDKN1B promoter hypermethylation

**Fig. 5:**
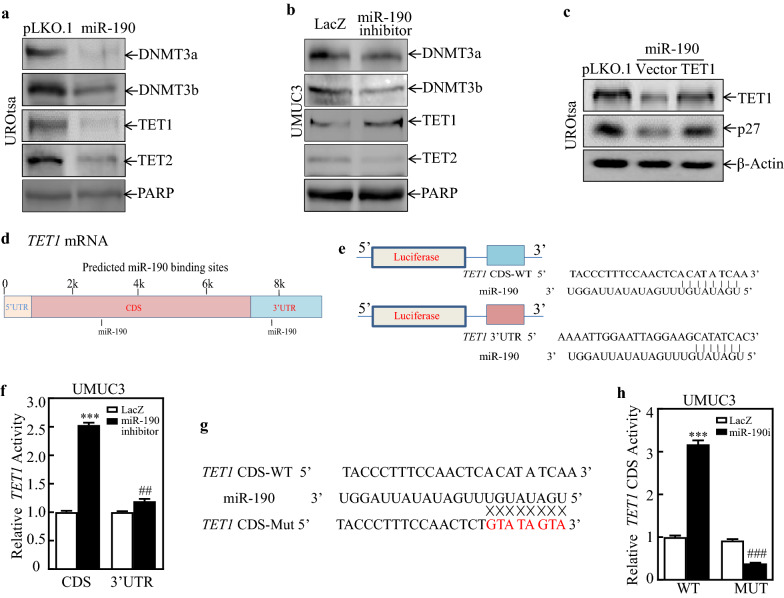
TET1 was a potential direct target gene of miR-190. **a**,** b** Cell lysates from indicated cells were evaluated for DNMT3a, DNMT3b, TETI, and TET2 expression. PARP was used as a protein loading control. **c** TET1 was transfected into UROtsa(miR-190)cells, and cell lysates from the indicated cells were subjected to western blot to analyze TET1 and p27 protein expression. β-Actin was used as a protein loading control. **d** Schematic diagram of CDS and 3’UTR of TET1 mRNA binding to miR-190. **e**,** f** Predicted miR-190 binding site region downstream of the firefly luciferase gene (pMIR-report Vector) was cloned (**e**), and pMIR-TET1-CDS were co-transfected with pRL-TK into the indicated UMUC3 (miR-190 inhibitor) and UMUC3(LacZ) transfectants. At 24 h after transfection, transfectants were extracted to assess luciferase activity (** f**); TK was used as the internal control. Each bar indicates the mean ± SD of three assays; *significant difference (*P < 0.05, **P < 0.01, ***P < 0.001; ^#^P < 0.05, ^##^P < 0.01, ^###^P < 0.001). **g** Potential miR-190 targeting sequences of the TET1 CDS were analyzed using TargetScan software. Schematics of the TET1 CDS luciferase reporter and its mutants (MUT) are shown. **h** WT and mutant pMIR-TET1-CDS reporters were co-transfected with pRL-TK into the indicated UMUC3 (miR-190 inhibitor) and UMUC3(LacZ) transfectants. At 24 h after transfection, transfectants were extracted to assess luciferase activity; TK was used as the internal control. Each bar indicates the mean ± SD of three assays; *significant difference (*P < 0.05, **P < 0.01, ***P < 0.001; ^#^P < 0.05, ^##^P < 0.01, ^###^P < 0.001)

DNMTs can transfer a group of methyl groups of the universal methyl donor *S*-adenosyl L-methionine to the 5′ position of cytosine residues in DNA [31]. The ten-eleven translocation (TET) enzymes (TET1, TET2, and TET3) are evolutionarily conserved dioxygenases that catalyze the conversion of 5-methyl-cytosine (5-mC) to 5-hydroxymethyl-cytosine (5-hmC) and promote DNA demethylation [32–34]. To investigate the potential role of any DNA methyltransferases (DNMTs) or demethylase in the hypermethylation of p27, DNMTs, and demethylase protein expression was evaluated among UROtsa (pLKO.1), UROtsa (miR-190), UMCU3 (LacZ), and UMUC3 (miR-190 inhibitor) cells. The results showed that miR-190 overexpression in UROtsa cells downregulated the expression of TET1, whereas miR-190 inhibition in UMUC3 cells upregulated the expression of the TET1 protein, suggesting that TET1 may be involved in miR-190-mediated *CDKN1B* hypermethylation (Fig. 5a, b). To explore this possibility, we stably transfected TET1 into UROtsa (miR-190) cells, as shown in Fig. 5c. TET1 overexpression markedly promoted p27 protein expression. Furthermore, we found that TET1 is also a direct target of miR-190. Bioinformatics analysis showed that both the CDS and 3′-UTR of *TET1* mRNA have miR-190 binding sites (Fig. 5d). To verify whether miR-190 acts on TET1 CDS or 3′-UTR, we cloned the TET1 CDS and 3′-UTR with the predicted miR-190 binding site region into the pMIR-report vector (Fig. 5e) and co-transfected with TK in UMUC3 (LacZ) and UMUC3 (miR-190 inhibitor) cells. UMUC3 (miR-190 inhibitor) cells transfected with pMIR-TET1-CDS exhibited an approximately 2.5-fold increase in luciferase activity compared to UMCU3 (LacZ) cells (Fig. 5f), whereas no obvious change was observed in pMIR-TET1-3′-UTR activity. These results indicate that miR-190 may inhibit TET1 by binding to the CDS of TET1 mRNA. We further site-mutated 8 nucleotides (UGAUAUGU) of the miR-190 binding site (Fig. 5g) and transfected the mutated constructs into UMUC3 (LacZ) and UMUC3 (miR-190 inhibitor) cells. As shown in Fig. 5h, the mutation of miR-190 binding sites abrogated the promoted effects of the miR-190 inhibitor on TET1-CDS luciferase reporter activity. Collectively, these data demonstrate that TET1 is a potential direct target gene of miR-190.

### C-Jun Mediates miR-190 up-regulation via promotion of *TLN2* transcription

**Fig. 6:**
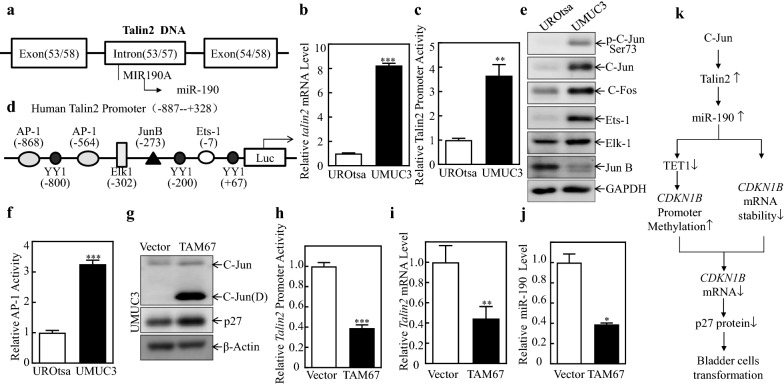
c-Jun plays an important role in the up-regulation of Talin2 mRNA and miR-190 level. **a **miR-190 is located in the intron region of the Talin2 gene. **b** Relative *Talin2* mRNA expression was detected in UROtsa and UMUC3 cells. *significant difference (*P < 0.05, **P < 0.01, ***P < 0.001). **c** The promoter activity of Talin2 was evaluated and compared between UROtsa and UMUC3 cells. *significant difference (*P < 0.05, **P < 0.01, ***P < 0.001). **d** Potential transcriptional factor-binding sites in the *Talin2* promoter region (– 887 to +328) analyzed by using the ALGGEN engine online. **e** The indicated stable transfectants were subjected to Western Blot to determine p-C-Jun Ser73, C-Jun, C-Fos, Ets-1, Elk-1, and JunB levels. GAPDH was used as a protein loading control. **f** Relative AP-1 activity was detected in UROtsa and UMUC3 cells. *significant difference (*P < 0.05, **P < 0.01, ***P < 0.001). **g** c-Jun dominant-negative mutant TAM67 and vector control plasmids were transfected into UMUC3 cells, and c-Jun dominant-negative mutant was identified by western blot. **h–j** Relative Talin2 promoter activity (**h**), Talin2 mRNA (**i**) and miR-190 level (**j**) detected in TAM67 was compared to that in their vector control cells. *significant difference (*P < 0.05, **P < 0.01, ***P < 0.001). **k** The proposed schematic for the mechanism underlying miR-190 contributes to Human Bladder Cell Transformation

miR-190 is conserved in mouse, rat, and human genomes and is located in the intron region of the *TLN2* gene (Fig. 6a) [35]. TLN2 can also regulate the expression of miR-190 [10, 36]. Therefore, we measured *TLN2* mRNA levels in UROtsa and UMUC3 cells (Fig. 6b), and the results showed that *TLN2* mRNA expression in UMUC3 cells was significantly higher than that in UROtsa cells. Then, the promoter activity of *TLN2* was evaluated and compared between UROtsa and UMUC3 cells. As shown in Fig. 6c, the promoter activity of TLN2 was significantly increased in UMUC3 cells, indicating increased transcription of TLN2/miR-190 in UMUC3 cells. Next, we performed a bioinformatics analysis of the *TLN2* promoter region and identified the potential binding sites of several transcription factors in the *TLN2* promoter region, including Ets1, YY1, AP-1, JunB, and Elk-1 (Fig. 6d). To define the specific transcription factor(s) involved in the regulation of *TLN2*, the protein expression of these transcription factors was examined in UROtsa and UMUC3 cells. As shown in Fig. 6e, c-Jun protein expression and phosphorylation at ser73 were both increased in UMUC3 cells, consistent with the alteration of relative AP-1 activity between UROtsa and UMUC3 cells (Fig. 6f). Therefore, the c-Jun dominant-negative mutant TAM67 was transfected into UMUC3 cells to determine the potential contribution of c-Jun to the activity of the *TLN2* promoter (Fig. 6g). Moreover, the ectopic expression of TAM67 successfully blocked *TLN2* promoter activity (Fig. 6h), *TLN2* mRNA (Fig. 6i), and miR-190 level (Fig. 6j) as well as increased p27 protein level (Fig. 6g), suggesting that c-Jun plays an important role in the upregulation of *TLN2* mRNA and miR-190 levels. Taken together, these results demonstrate that c-Jun promotes TLN2/miR-190 transcription, thereby upregulating miR-190 expression in human BC cells. Our results conclusively indicate that c-Jun upregulates miR-190 to promote bladder cell transformation by inhibiting *CDKN1B* mRNA stability. In addition, miR-190 targets TET1 CDS to inhibit TET1 protein expression and attenuate the demethylation of the *CDKN1B* promoter, thereby inhibiting *CDKN1B* mRNA transcription, as summarized in Fig. 6k.

## Discussion

Many studies have shown that miR-190, as an oncogene or tumor suppressor gene, is involved in regulating the proliferation and metastasis of tumor cells [12, 13]. However, few studies have investigated the role and mechanism of miR-190 in BC. We found that miR-190 was overexpressed in human BC tissues and contributed to bladder cell transformation. A further study found that p27 downregulation plays a crucial role in miR-190, inducing S phase in the cell cycles of BC cells. The cyclin-dependent kinase (Cdk) inhibitor p27 (also known as KIP1) was discovered as a mediator of growth arrest [37]. A previous study showed that p27 impedes cell cycle progression by inhibiting cyclin-dependent kinases (CDKs) by transforming growth factor β (TGF-β) [38]. p27 expression is reduced in various tumors including lung [39], head and neck [40], colorectal [41], and ovarian cancers [42], and is associated with prognosis. The expression of p27 in cells is strictly regulated, including transcription [43], protein translation [44], nuclear to cytoplasmic transport [45], and protein degradation [46], among others. Our results showed that miR-190 simultaneously downregulates the p27 protein by directly acting on the p27 mRNA and TET1-mediated hypermethylation level to suppress transcription. Mechanistic research showed that miR-190 mainly downregulated p27 in terms of mRNA levels, which can inhibit p27 mRNA stability by binding to p27 mRNA 3’-UTR. On the other hand, miR-190 simultaneously inhibits p27 transcription by upregulating p27 promoter methylation in human BC cells. The methylation level in the promoter region is mainly mediated by DNMTs and demethylases. DNMTs can transfer a group of methyl groups of the universal methyl donor S-adenosyl L-methionine to the 5 position of cytosine residues in DNA. The TET enzymes (TET1, TET2, and TET3) catalyze the conversion of 5-mC to 5-hmC and promote DNA demethylation. The TET enzymes family are Fe^2+^ and 2-oxoglutarate-dependent dioxygenases, and TET1 and TET3 contain a CXXC zinc finger domain at their amino-terminus, which is known to bind CpG sequences [47]. Our results suggest that TET1 plays a key role in mediating the hypermethylation level of the p27 promoter. Furthermore, miR-190 downregulated TET1 by binding its CDS region, as well as mediated p27 promoter hypermethylation. Therefore, we conclude that miR-190 simultaneously downregulates the p27 protein by directly acting on p27 mRNA and TET1-mediated hypermethylation levels to suppress transcription.

The expression of miR-190 varies in different tumors, and this expression is increased in BC, but the mechanism behind this remain unclear. c-Jun is a proto-oncoprotein that can heterodimerize with c-Fos to form the activator protein-1 (AP-1) [48]. The absence of c-Jun results in elevated expression of the tumor suppressor gene [49]. Previous reports have shown that c-Jun enhancement of androgen receptor transactivation is associated with prostate cancer cell proliferation [50]. Our data indicate that the transcription of TLN2/miR-190 in UMUC3 cells was increased. c-Jun protein and c-Jun phosphorylation (ser73) levels were increased in UMUC3 cells. Furthermore, ectopic expression of TAM67 successfully blocked TLN2 promoter activity, TLN2 mRNA, and miR-190 levels as well as increased p27 protein levels. Therefore, we concluded that c-Jun promotes TLN2/miR-190 transcription, thereby upregulating miR-190 expression in human BC cells.

## Conclusions

In summary, the current study showed for the first time that miR-190 is upregulated in human BC tissues and contributes to bladder cell transformation. Moreover, c-Jun mediated miR-190 upregulation via promotion of TLN2 transcription. miRNAs play biological roles by modulating target gene expression through binding to the 3’-UTR of target genes or by up-regulating p27 promoter methylation to cause mRNA stability alteration or protein translation suppression. Taken together, these results indicate that miR-190 is a critical cancer-promoting molecule in BC and that miR-190 and its target genes may serve as potential targets for early diagnosis and/or as targets for the treatment of BC patients.

## Data Availability

The datasets generated and analyzed during the current study are available from the corresponding author on reasonable request.

## Abbreviations

Act D: Actinomycin D
BC: Bladder cancer
UC: Urothelial carcinomas
NMIBC: Non-muscle invasive tumors
MIBC: Muscle invasive tumors
UTR: Untranslated region
DNMT: DNA methyltransferase
